# The SAPIENS 3D-printed temporal bone model: a real tool for advanced otologic surgery education

**DOI:** 10.1007/s00405-024-09199-3

**Published:** 2025-02-17

**Authors:** Giannicola Iannella, Annalisa Pace, Antonio Greco, Armando De Virgilio, Mario Giuseppe Bellizzi, Enrica Croce, Jerome R. Lechien, Antonino Maniaci, Salvatore Cocuzza, Federico Maria Gioacchini, Massimo Re, Andrea Collettini, Lodovica Gatti, Tiziano Perrone, François Simon, Stéphane Gargula, Giuseppe Magliulo

**Affiliations:** 1 Department of ‘Organi di Senso’, University “Sapienza”, Viale dell’Università 33, Rome, 00185 Italy; 2 Faculty of Medicine and Pharmacy, University of Mons (UMons), Mons, Belgium; 3 Department of Otolaryngology, Kore University, Enna, Italy; 4 Department of Medical, Surgical Sciences and Advanced Technologies G.F. Ingrassia, University of Catania, Catania, Italy; 5 Ear, Nose, and Throat Unit, Department of Clinical and Molecular Sciences, Polytechnic University of Marche, Ancona, Italy; 6 Department of Otolaryngology, Civil Hospital of Alghero, Alghero, Italy; 7 Department of Pediatric Otolaryngology, Necker-Sick Children’s Hospital, AP-HP-University of Paris, Paris, France; 8 Department of Otolaryngology, Hôpital Fondation Adolphe de Rothschild, Paris, France

**Keywords:** 3D-printed temporal bone, Temporal bone, Dissection, Ear surgery, Training, Otology

## Abstract

**Objective:**

Our study focused on the development and evaluation of the SAPIENS (Specific Anatomical Printed-3D-model In Education and New Surgical Simulations) as a valid tool for otologic surgical education.

**Methods:**

Twenty junior otolaryngologist surgeons in training were enrolled in the study. Each participant was invited to perform dissection of three different temporal bones. 1)Transparent 3-D printed model; 2)Opaque 3-D model; 3)fresh frozen human temporal bone. Following their drilling experience, participants answered to two specific questionnaires. The first was a questionnaire developed by Mowry et al. to evaluate 3D models in its general characteristics of anatomy and dissection, while the second one was a questionnaire specifically designed to compare the 3-D printed models with the human fresh frozen temporal bone.

**Results:**

The average total score of the questionnaire was calculated as 53.2/61 in transparent 3-D model and 55.4/61 in the opaque 3-D model. These values indicate that the 3D printed models closely resemble the human TB in terms of anatomy and dissection. Comparisons of the 3D model and human TB were rated as very similar in all surgical steps. The total score was 4/5 in the transparent 3-D model and 4.2/5 in the opaque 3-D model.

**Conclusion:**

We have designed and developed a 3D-printed model of the temporal bone that closely resembles the human temporal bone. The SAPIENS 3-D printed temporal bone model could be considered a valuable tool for advancing oto-surgical education due to its similarity to the human temporal bone in terms of anatomy and dissection.

## Introduction

In the realm of surgical education, advancements in technology continuously reshape the landscape, offering innovative tools to enhance learning experiences and skill development. One such breakthrough is the use of three-dimensional (3D) printing technology to create anatomically accurate models capable of simulating temporal bone dissection, providing a valid platform for surgical training [1–6].

The complexity of temporal bone surgical techniques requires not only a thorough understanding of the 3D spatial relationships of structures within the temporal bone but also exceptional surgical dexterity in confined spaces. Given this, traditional teaching methods, which rely solely on limited cadaveric specimens and two-dimensional illustrations, may not sufficiently prepare surgeons for the challenges encountered during otologic surgery [1–12].

3D-printed temporal bone models faithfully replicate intricate anatomy, allowing surgeons to practice various procedures in a realistic surgical environment. These models provide tactile feedback that mimics the feel of bone and soft tissues, thereby enhancing the realism of the training experience [14–24]. Furthermore, they can be customized to simulate specific pathologies or surgical scenarios, enabling trainees to practice a wide range of procedures. Surgeons can use 3D-printed temporal bone models to plan and simulate complex surgeries, optimizing surgical outcomes and reducing the risk of complications [18–32].

Additionally, incorporating 3D-printed temporal bone models into surgical education has been shown to accelerate the learning curve for trainees. Preliminary studies have demonstrated that trainees exposed to simulation-based training using 3D-printed models exhibit improved procedural skills and greater confidence compared to those trained using conventional methods. By providing a safe and controlled environment for otologic practice, these models allow trainees to refine their surgical skills and techniques without the pressure of operating on live patients [17–32].

Recently, our study group developed a new 3D temporal bone model called SAPIENS (Specific Anatomical Printed 3D Model In Education and New Surgical Simulations), which closely resembles the human temporal bone [31]. In a preliminary study published by Iannella et al. [33], this 3D model was introduced and evaluated for its overall replication of the tympano-mastoid structures of the temporal bone. The model was considered to exhibit remarkable similarity to the human temporal bone in terms of middle and inner ear anatomy, as evaluated by five experienced otologic surgeons. It enables surgical dissection of the middle ear and mastoid with an excellent degree of similarity to dissections performed on cadaveric temporal bones [33].

In this subsequent study, we evaluated the SAPIENS model for its effectiveness in teaching otologic surgery to young ENT surgeons. This paper aims to explore the potential of 3D-printed temporal bone models in supporting otologic surgical education and learning curves, with a focus on their utility, benefits, and impact on improving surgical skills.

## Materials and methods

The development and production of the described 3D temporal bone model took place between June 2022 and October 2023 at the Department of Sense Organs, Sapienza University of Rome. The project received IRB exemption from the Institutional Review Board at Sapienza University of Rome. A multidisciplinary team, including radiologists, software engineers, ENT specialists, and 3D-printing experts, collaborated on this unique 3D-printed temporal bone (TB) model.

Every developmental step, including the pre-printing, printing, and post-printing phases involved in creating the SAPIENS 3D-printed temporal bone model, has been documented in the initial study by Iannella et al. [33].

**Fig. 1:**
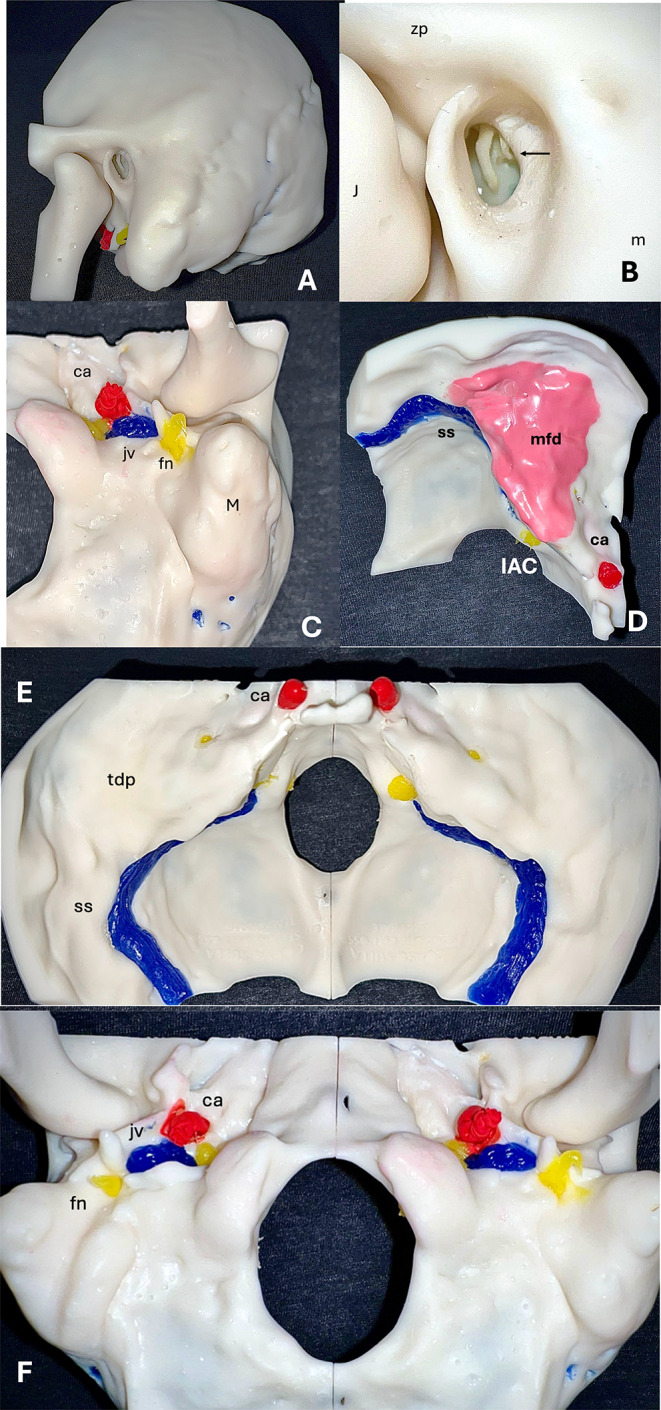
Final 3D-printed ‘SAPIENS’ temporal bone model; (**A**) Lateral coronal view; the external auditory canal, mastoid bone, stylomastoid process, and zygomatic process are visible; the facial nerve (yellow) and carotid artery (red) are marked. (**B**) Magnified lateral coronal view; the malleus handle is clearly visible, with an arrow indicating the incudo-stapedial joint; the stapes superstructures are visible from the external auditory canal (EAC). (**C**) External skull base and temporal bone axial view; the mastoid process (M) is visible; the emergence of the facial nerve (fn) from the skull base, marked in yellow, the carotid artery (ca) marked in red, and the jugular vein (gv) marked in blue, are visible. (**D**) Axial view of the internal skull base and petrous bone; the temporal bone plate is marked in pink, and a layer of soft tissue simulating the dura of the middle cranial fossa during dissection is placed. The sigmoid sinus (SS) and carotid artery (ca) are visible; the facial nerve in the Internal Auditory Canal (IAC) is visible. (**E**) Internal skull base and petrous bone in a bilateral reconstruction. The sigmoid sinus (SS) and carotid artery (ca) are visible; the facial nerve in the IAC is visible. (**F**) Inferior view of the external skull base and petrous bone in a bilateral reconstruction; the emergence of the facial nerve (fn) from the skull base, marked in yellow, the carotid artery (ca) marked in red, and the jugular vein (gv) marked in blue, are visible

The final SAPIENS 3D-printed model of the temporal bone is shown in Fig. 1 (opaque 3D-printed model), demonstrating remarkable similarity to the human temporal bone in terms of middle and inner ear anatomy.

**Fig. 2:**
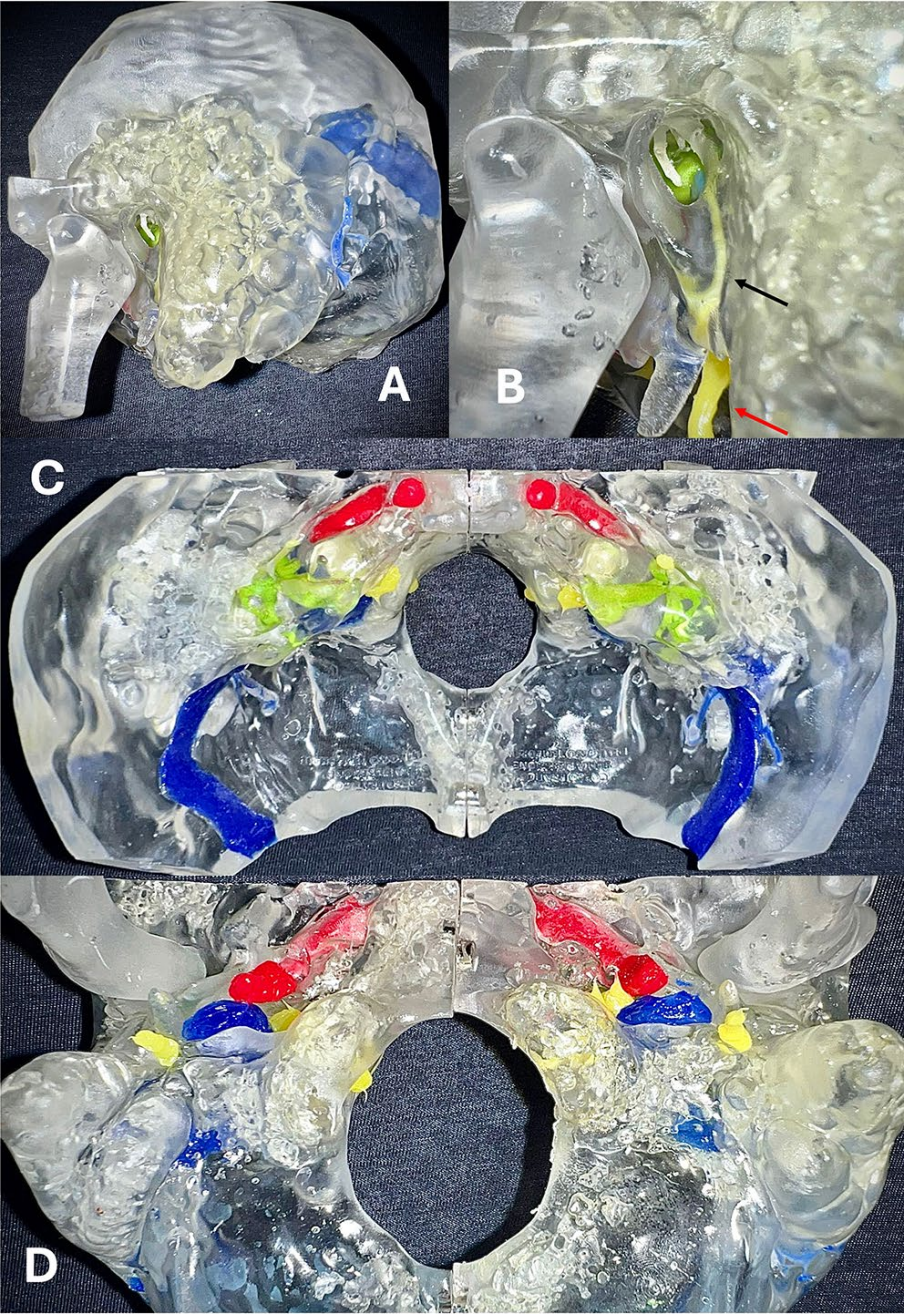
Transparent 3D-printed ‘SAPIENS’ temporal bone model; transparent model created using colorless resin for the temporal bone and different-colored silicones to mark the inner ear structures. (**A**) Lateral coronal view; the external auditory canal, mastoid bone, stylomastoid process, and zygomatic process are visible; the malleus handle is well visible, as is the emergence of the facial nerve (fn) from the skull base, marked in yellow. (**B**) Magnified lateral coronal view; the malleus handle is visible, as well as the facial nerve; the red arrow indicates the facial nerve, and the black arrow points to the corda tympani nerve; the incudo-stapedial joint is visible from the EAC. (**C**) Transparent model of the internal skull base and petrous bone in bilateral reconstruction. The sigmoid sinus (blue), carotid artery (red), and facial nerve (yellow) in the IAC are visible. The superior semicircular canal and the apical turn of the cochlea (green) are also visible. (**D**) Transparent model of the external skull base and petrous bone in bilateral reconstruction; the emergence of the facial nerve (fn) from the skull base, marked in yellow, the carotid artery (ca), marked in red, and the jugular vein (gv), marked in blue, are visible

To facilitate the teaching and understanding of temporal bone anatomy and to highlight hidden structures, we also developed a transparent 3D-printed model. This model was made using a colorless resin, with inner ear structures (cochlea, semicircular canals, vestibule) and lateral skull base components (tegmen tympani, carotid artery, sigmoid sinus, and jugular bulb) represented and visible through the precise injection of differently colored silicones (Fig. 2).

### Evaluation to use of the 3D printed temporal bone model in surgical training

Twenty-five junior otolaryngology surgeons in training (ENT residents) volunteered to participate in the second phase of this study to assess the effectiveness of the 3D-printed temporal bone model in otologic surgical training.

Participants were selected among ENT residents with limited knowledge of temporal bone (TB) anatomy and minimal experience with TB dissection.

The validation process for the 3D TB model involved comparative dissections between the artificial 3D model and a fresh-frozen human temporal bone from a cadaver.

Each participant performed dissections on three different temporal bones over three separate days:

Day One: Transparent 3D-printed bone model.

Day Two: Opaque 3D-printed bone model.

Day Three: Fresh-frozen human temporal bone.

All dissections were conducted in the temporal bone laboratory at Sapienza University of Rome. Residents were provided with all necessary equipment, including tools typically used in an operating theater (microscope, micro-drills, irrigation-suction system), as well as personal protective equipment (gowns, gloves, masks, and eye protection).

### The simulated surgical steps were consistent across all three anatomical preparations

**Fig. 3:**
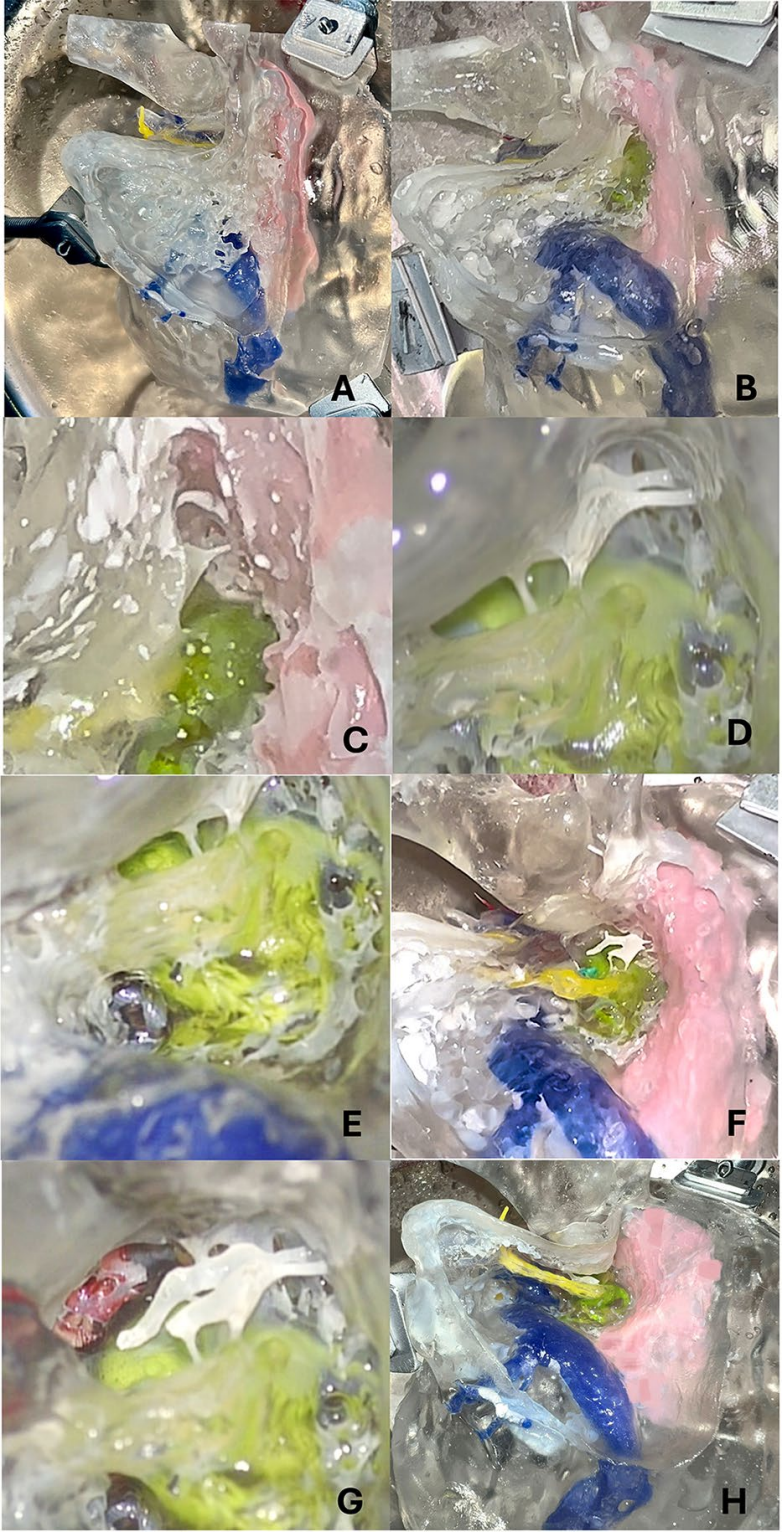
Surgical dissection of the transparent 3D-printed temporal bone model (lateral view). Dissection was performed by a young surgeon in training (ENT resident) to demonstrate the effective use of this 3D-printed petrous bone model in otologic surgery training. (**A**) Cortical mastoidectomy, with well-represented mastoid bone pneumatization. (**B**) Mastoidectomy with sigmoid sinus (blue) and temporal dura plate (pink) clearly identified. (**C**) Mastoidectomy with epitympanotomy in a magnified view; the body of the incus with its ligament is visible; facial nerve (yellow) and lateral semicircular canal (green) are easily identified in transparency. (**D**) Posterior tympanotomy; short and long processes of the incus and the head of the malleus are visible (s); lateral semicircular canal (green) and facial nerve (yellow) are visible in transparency. (**E**) Magnified view of the posterior tympanotomy; round window (green) is visible. (**F**) Removal of the posterior part of the external auditory canal. The incudo-stapedial joint, cochlea (dark green), facial nerve (yellow), and labyrinthine structures (green) are visible in transparency. (**G**) Identification of the internal carotid artery (red). (**H**) Skeletonized facial nerve and dissection of the under-facial recess with access to the hypotympanic space and identification of the jugular bulb

**Fig. 4:**
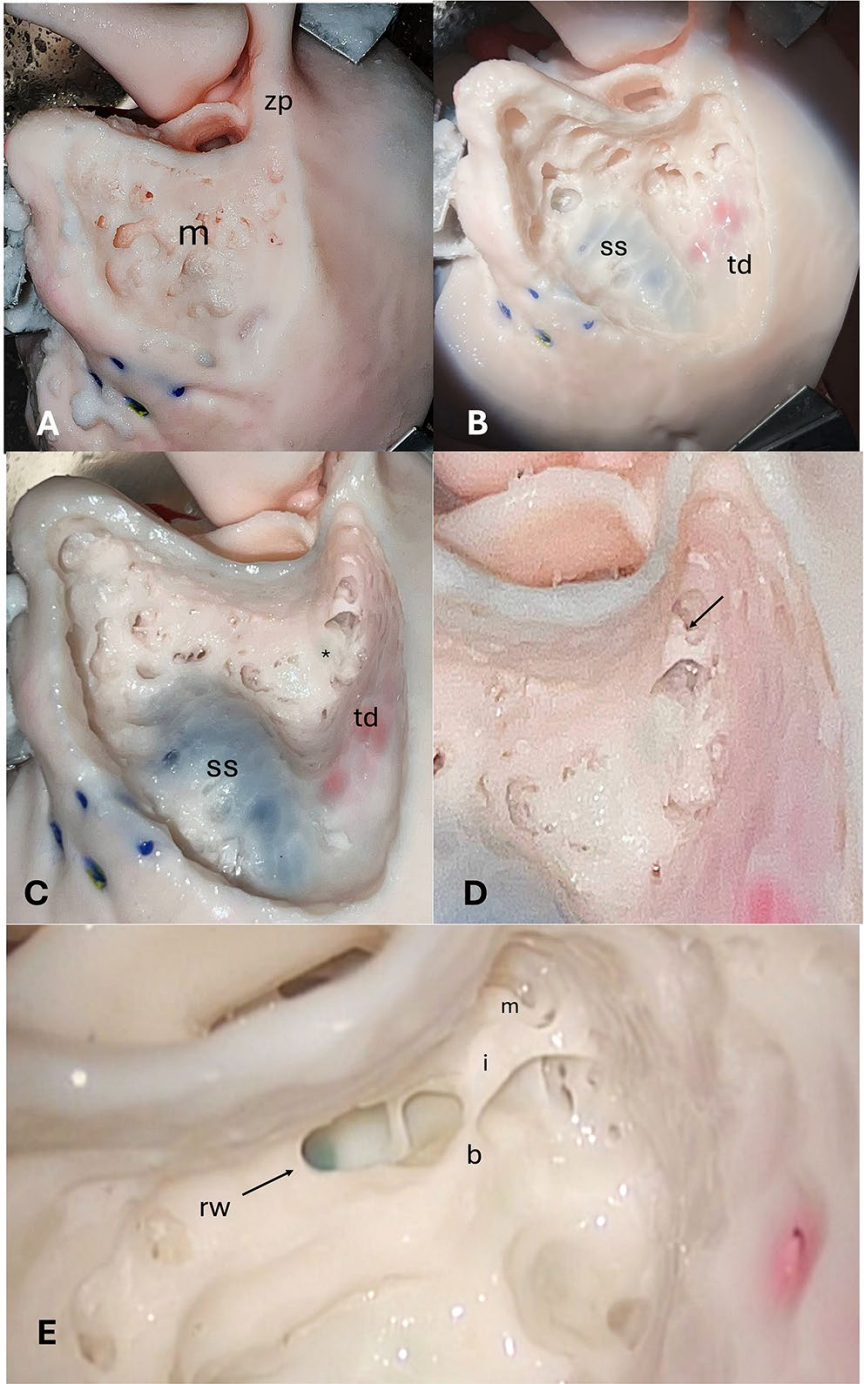
Surgical dissection of the opaque 3D-printed temporal bone model (lateral view). (**A**) Cortical mastoidectomy a with well-represented mastoid bone pneumatization. (**B**) Mastoidectomy with sigmoid sinus (blue color) and temporal dura plate (pink color) identified. (**C**) Mastoidectomy with epitympanotomy. Body of the incus is visible. (**D**) Mastoidectomy with epitympanotomy in a magnified view. Body of the incus and head of the malleus are visible. (**E**) Posterior tympanotomy; (m) malleus, i (incus), b (buttress). Round window (rw) is well visible and marked in dark green color

**Fig. 5:**
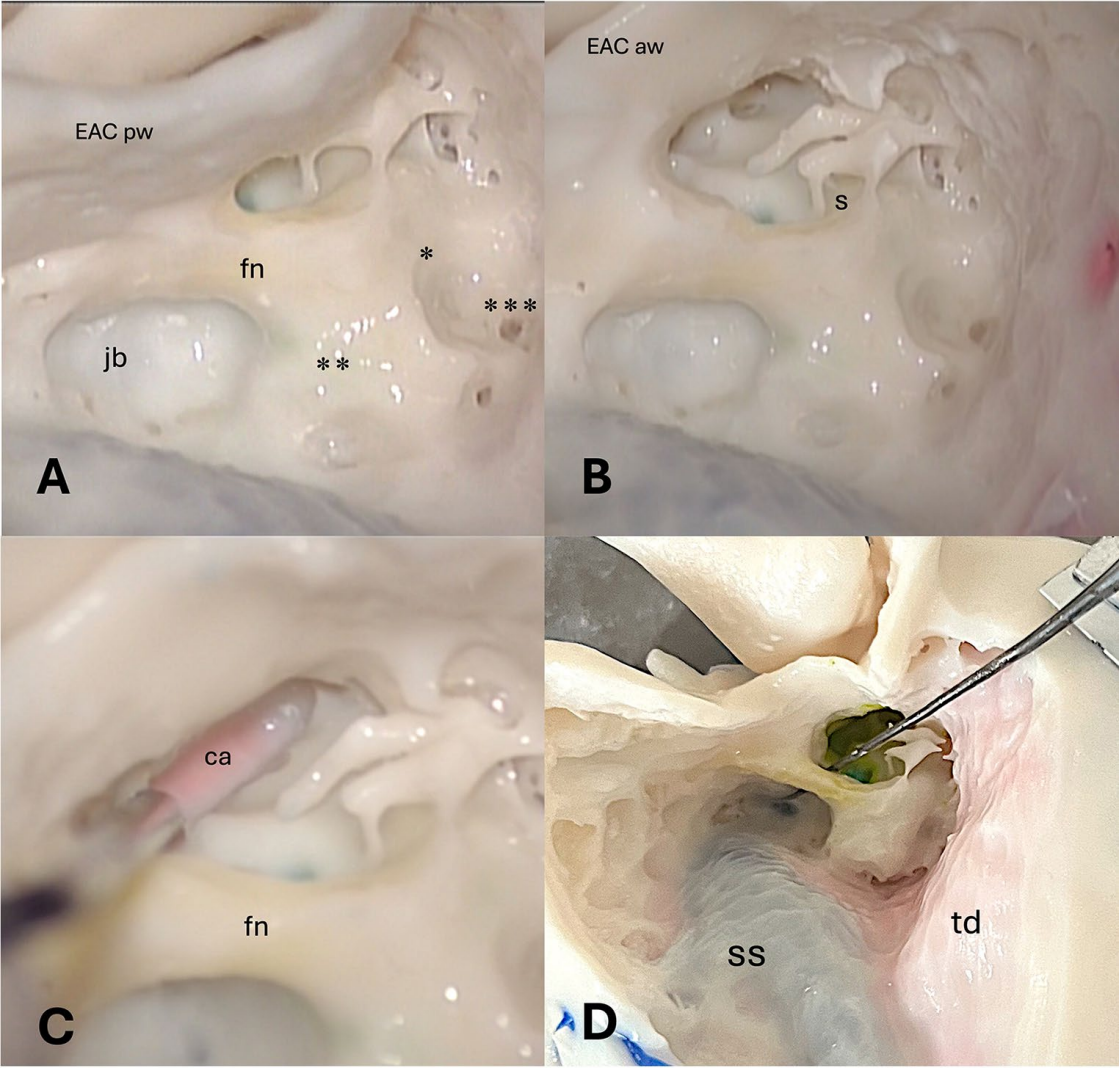
Surgical dissection of the opaque 3-D printed temporal bone model (lateral view). (**A**) Complete mastoidectomy with facial nerve (fn) skeletonization in the mastoid segment. Posterior wall of external auditory canal (EACpw); jugular bulb (jb); facial nerve (fn); * lateral semicircular canal; ** posterior semicircular canal; *** superior semicircular canal. (**B**) Removal of the posterior part of the external auditory canal. The incudo-stapedial joint and facial nerve (yellow), round window (dark green) are clearly visible. Anterior wall of the external auditory canal (EACaw); stapes (s). (**C**) Identification of the internal carotid artery (red); facial nerve (fn). (**D**) Skeletonized facial nerve and dissection of the under-facial recess with access to the hypotympanic space (specimen inside) and identification of the jugular bulb. Sigmoid sinus (SS); temporal dura plate (td)

- Step I - Simple mastoidectomy (Figs. 3A-B and 4A-C).
- Step II - Epy-tympanotomy with identification of the short process of the incus and incudo-malleolar joint (Figs. 3C and 4C-D).
- Step III step - Posterior tympanotomy and identification of the round window (Figs. 3D-E and 4E).
- Step IV step - Hypotympanic dissection and identification of the jugular bulb (Figs. 3E and 5A).
- Step V step - Canal wall down technique (Figs. 3F and 5B).
- Step VI step - Identification of the internal carotid artery (Figs. 3G and 5C).
- Step VII – Facial nerve dissection (Figs. 3H and 5C-D).

The time taken by each resident to perform each step of the dissection on each model was recorded, and the total dissection time was evaluated.

To assess the performance of both 3D-printed models in otologic surgical dissection and to compare them with fresh-frozen human TBs, participants completed two specific questionnaires (Table 1) following their drilling experience:

**Table 1:** Evaluation of the SAPIENS 3D printed model in surgical dissection

**Mowry questionnaire evaluating temporal bone dissection of the 3D printed models.** Fourteen questions were presented on a 4-point Likert scale (1- being far from reality, to 4- very real). The final question inquired if the model could be used as a simulator for surgical practice (utilizing a 5-point scale in this case) Average value of twenty-five resident answers has been reported								
				Transparent 3-D printed ‘SAPIENS’ temporal bone model (average value)	Opaque 3-D printed ‘SAPIENS’ temporal bone model (average value)			
1.	External contour			3.5	3.8			
2.	Texture of the drilled plastic mastoid architecture			2.6	3.6			
3.	Anatomy of the antrum (incus pointer)			3.6	3.7			
4.	Tegmen contour			3.6	3.8			
5.	Otic capsule contour and density			3.2	3.8			
6.	Sigmoid sinus contour			4	3.8			
7.	Did the model recreate the change in pitch heard during in vivo temporal bone surgery?			3.2	3.7			
8.	How is the reflectivity of the model? (how much the light penetrated the material to allow visualization of the deeper structures)			4	3.6			
9.	Absence of odor production when dissected			4	4			
10.	Dust formation during dissection			3.2	3.8			
11.	Supporting material absence			4	4			
12.	Facial nerve			4	3.8			
13.	Facial nerve recess			4	3.8			
14.	Overall, do you feel the best bone accurately recreated a model for cortical mastoidectomy?			4	3.6			
15.	Do you feel this model can be used as a simulator for surgical practice?			4	4			
	Total value			**53.2**	**55.4**			
**Comparative questionnaire between SAPIENS 3D printed model and human cadaver temporal bones** Fourteen questions were presented on a 5-point Likert scale (1- being far from similarity with human fresh frozen temporal bone, to 5- very similar with human fresh frozen temporal bone) Average value of twenty-five resident answers has been reported.								
						Transparent 3-D printed ‘SAPIENS’ temporal bone model		Opaque 3-D printed ‘SAPIENS’ temporal bone model
1		Please rate the general temporal bone anatomical structures details of the 3-d printed TB model in comparison with a human TB from cadaver.				3.9		4.17
2		Please rate the likeness of the mastoid region drilling dissection (tactile feel – sound – visual appearance) of the 3D-printed TB model in comparison with the human temporal bone from cadaver				3.2		4.17
3		Please rate the likeness of the 3D-printed TB model (tactile feel – sound – visual appearance) in comparison with the human cadaveric temporal bone in tegmen tympani and sigmoid sinus identification and skeletonization				4.8		4.50
4		Please rate the likeness of the dissection (tactile feel – sound – visual appearance) of the Epitympanic region (superior tympanotomy) of the 3D-printed TB model in comparison with the human temporal bone from cadaver				4.1		4.67
5		Please rate the likeness of the dissection (tactile feel – sound – visual appearance) of the posterior tympanotomy of the 3D-printed TB model in comparison with the human cadaveric temporal bone				4.5		4.67
6		Please rate the likeness of the dissection (tactile feel – sound – visual appearance) of the ossicular chain of the 3D-printed TB model in comparison with the human cadaveric temporal bone				3.2		4.00
7		Please rate the likeness of the posterior wall drilling dissection (tactile feel – sound – visual appearance) of the 3D-printed TB model in comparison with the human cadaveric temporal bone				4.6		4.67
8		Please rate the likeness of the ipo-tympanic drilling dissection (tactile feel – sound – visual appearance) of the 3D-printed TB model in comparison with the human cadaveric temporal bone				4.6		4.50
9		Please rate the likeness of the carotid artery drilling dissection (tactile feel – sound – visual appearance) of the 3D-printed TB model in comparison with the human cadaveric temporal bone				4.8		4.50
10		Please rate the likeness of the facial nerve dissection (tactile feel – sound – visual appearance) of the 3D-printed TB model in comparison with the human cadaveric temporal bone				4.8		4.5
		**Total value**				4		4.2
**Surgical dissection times**								
			3d printed ‘SAPIENS’ temporal bone model			Transparent 3d printed ‘SAPIENS’ temporal bone model	Fresh frozen temporal bone model	
Step I - Simple mastoidectomy			41.6			38.5	30.6	
Step II - Epy tympanotomy with identification of the short process of the incus and incudo-malleolar joint.			35.7			33.5	30.8	
Step III step - Posterior tympanotomy and identification of the round window			35.5			32.7	28.2	
Step IV step - Hypotympanic dissection and identification of the jugular bulb			45.4			30.4	25.3	
Step V step - Canal wall down technique			40.1			32.2	28.7	
Step VI step - Identification of the internal carotid artery			30.6			23.8	16.6	
Step VII – Facial nerve dissection			48.5			38.4	27.6	
Total Time Of Specimen Dissection			277.4			229.5	187.8	

Questionnaire 1: Developed by Mowry et al. [15], this questionnaire was specifically designed to evaluate 3D temporal bone models. It had been previously utilized in a multi-institutional study comparing various TB models as well as in other clinical studies assessing new 3D-printed temporal bone models. The questionnaire consisted of 15 queries exploring the characteristics of the 3D-printed TB model, middle ear and mastoid anatomy, and the reproducibility of various surgical dissection steps. Fourteen questions were presented on a 4-point Likert scale (1 = far from reality, 4 = very realistic). The final question asked whether the model could be used as a simulator for surgical practice, utilizing a 5-point scale for this evaluation. The maximum score achievable was 61, and a total score above 40 was considered indicative of a 3D-printed model that closely resembles the human TB in terms of anatomy and dissection.

Questionnaire 2: This was specifically designed to compare the two SAPIENS 3D-printed models with fresh-frozen human temporal bones (currently regarded as the gold standard for temporal bone dissection). It consisted of 10 questions investigating the reproducibility and similarity of different surgical dissection steps, using a 5-point Likert scale (1 = far from similar to human fresh-frozen temporal bone, 5 = very similar to human fresh-frozen temporal bone).

## Results

### Participants in the study

All participants enrolled in the study were young ENT surgeons from various centers in their first or second year of residency. Eighteen out of the 20 participants had never performed a mastoid-tympanic dissection, either in vivo or on fresh-frozen temporal bone specimens. The remaining two participants had performed only one previous cadaveric temporal bone mastoid-tympanic dissection. Therefore, the participant group was considered homogeneous in terms of their clinical and practical knowledge of mastoid-tympanic anatomy and dissection.

### 3D printed TB model evaluation

The evaluation of the 3D-printed temporal bone (TB) models is presented in Table 1. The average total score on the Mowry questionnaire was 53.2 for the transparent TB model and 55.4 for the opaque TB model. These scores indicate that the 3D-printed models closely resemble the human TB in terms of anatomy and dissection. Sub-scores, based on responses from different surgeons, are also reported in Table 1. Notable scores were achieved in the following areas: sigmoid sinus (Q-6 average value of 4/4 and 3.8/4), Facial nerve (Q-12 average value of 4/4 and 3.8/4), cortical mastoidectomy simulation (Q-14 average value of 4/4 and 3.6/4), and the use of the model for simulating surgical practice (Q-15 average value of 4/4 and 4/4).

### Comparison: 3D printed TB models vs. fresh-frozen temporal bone

Results of the comparative dissection analysis between the 3D-printed models and human cadaver temporal bones are reported in Table 1. The 3D models were rated as very similar to the human TB across all surgical steps. The total scores were 4/5 for the transparent TB model and 4.2/5 for the opaque TB model.

In the sub-analysis of the transparent 3D-printed model vs. fresh-frozen TB, notable findings include:

- The average rating for the temporal bone details (Q1) was 3.9.
- The identification and skeletonization of the tegmen tympani and sigmoid sinus (Q3) as the posterior tympanotomy (Q5) reported very high values of 4.8 and 4.5 respectively.
- Most importantly facial nerve dissection reached an average value of 4.8, indicating a very similar dissection.
- However, the mastoid dissection (Q2) and ossicular chain (Q6) reproduced in the 3D transparent model showed a lower degree of similarity with the cadaveric temporal bone, with values of 3.2 each.
- In the sub-analysis of the opaque 3D printed model vs. fresh frozen TB, key findings include:
- An average rating (Q1) of 4.17.
- The mastoid region dissection (Q2) was considered very similar to the human temporal bone, as well as the identification and skeletonization of the tegmen tympani and sigmoid sinus (Q3), with reported values of 4.17 and 4.5 respectively.
- The Epitympanic region dissection (Q4) reported a value of 4.67, and the execution of the posterior tympanotomy (Q5) scored 4.67.
- The ossicular chain (Q6) reproduced in the 3D model achieved a slightly better value of 4.
- Canal wall down (Q7) was well reproduced with a value of 4.67, while the facial nerve dissection (Q10) reached an average value of 4.5.

Table 1 displays the average dissection times of each surgical step and the total time used to complete the dissection, for each of the three models. Total dissection time was 277.4 min for the transparent 3D printed model, 229.5 min for the opaque 3D-printed TB model, and 187.8 min for the fresh frozen temporal bone, respectively. Statistical difference emerged between the average dissection time in the transparent 3D printed TB model and the fresh frozen temporal bone (*p* = 0.002). The reduction in dissection time across the three models indicated improved knowledge of temporal bone anatomy and enhancement in surgical skills.

### 3D-temporal models - surgeon feedback

The 3D-printed temporal bone models were consistently regarded as highly similar to human temporal bones for dissection practice.

The **transparent model** was considered ideal for inexperienced surgeons, as it provided a clear visualization of the marked anatomical structures. This feature facilitated an accurate understanding of the spatial arrangement, enabling more precise dissection and greater familiarity with the anatomy in preparation for working with the opaque and fresh-frozen models.

In the **opaque model**, the mastoid displayed well-pneumatized and compact bone coverage. The texture of the mastoid architecture, closely resembling that of a human temporal bone, allowed for realistic drilling. The **sigmoid sinus**, marked with blue dye, was distinctly visible during mastoidectomy. The **tegmen tympani**, highlighted with pink dye, was easily identifiable during mastoid dissection, providing accurate guidance for drilling while respecting the dura’s direction.

Key anatomical landmarks were effectively represented:

- The **ossicular chain**, **round window**, **promontory**, and the **Fallopian canal** with the facial nerve (injected with yellow-colored silicone) were well-rendered and could be dissected accurately.
- The **internal carotid artery** (stained red) and **jugular bulb** (stained blue) were clearly identifiable during dissection.
- The **semicircular canals**, **vestibule**, and **cochlea**, which featured increased resin density to simulate the compact nature of labyrinthine bone, were marked in green. These structures appeared hollow during dissection, enhancing the realism of the practice.

Notably, the model demonstrated resilience during dissection, showing no abnormal fractures. Additionally, it provided a tactile feel, sound, and visual appearance that closely mimicked cadaveric temporal bones, contributing to an authentic surgical training experience.

## Discussion

Three-dimensional printing of temporal bones represents a groundbreaking innovation in surgical education methods. Numerous investigators have independently developed 3D-printed temporal bone (TB) models for both surgical training and preoperative simulation of complex cases [13–33].

Mowry et al. [15] conducted a comparison of 3D-printed TB models, evaluating a total of 12 models in a dissection laboratory. Each model was scored based on its performance during dissection. The authors reported that a total score exceeding 40 indicated a 3D-printed model with strong anatomical and dissection fidelity to the human TB.

Frithioff et al. [31] assessed the use of a low-cost 3D-printed TB model for mastoidectomy training with 18 novice otorhinolaryngology residents. The study reported positive results, highlighting the model’s suitability for surgical training. Similarly, Chien et al. [30] validated the use of 3D-printed TB models as alternative training tools for otologic surgery. Participants found these models to be anatomically realistic and comparable to cadaveric TBs. Recently, Gadaleta et al. [18] developed a very realistic temporal bone model that was deemed appropriate in surgical train dissection by 10 ENT residents in training. Participants found this 3D model to be like cadaveric temporal bones, particularly in safety, overall anatomy, and mastoid dissection.

Our research group recently developed the SAPIENS, a 3D-printed temporal bone (TB) model designed to provide a high-fidelity reproduction of human temporal bone structures [33]. In the initial study by Iannella et al. [33], the design and production phases of this 3D model were described in detail. The SAPIENS model was evaluated using Mowry et al.‘s questionnaire [16] by five experienced ENT surgeons, achieving an average total score of 49.4 ± 1.8 out of 61. This score indicates a model highly comparable to human TB anatomy.

The SAPIENS 3D-printed TB model demonstrated a high degree of anatomical accuracy, effectively replicating structures of the middle and inner ear. Key components, including the sigmoid sinus, tegmen tympani, ossicular chain, round window, promontory, Fallopian canal with the facial nerve, internal carotid artery, and jugular bulb, were faithfully reproduced. Furthermore, the model enabled middle ear and mastoid surgical dissection with excellent similarity to procedures performed on cadaveric TBs, greatly enhancing its educational value [33–38].

In a subsequent study, we assessed the SAPIENS model’s utility in training and developing surgical skills among young ENT surgeons. This evaluation involved two different versions of the SAPIENS model (transparent and opaque) and 20 ENT residents. The previously mentioned Mowry et al. questionnaire [16] was utilized to assess the overall appearance, tactile experience, and anatomical fidelity of the 3D-printed models. The transparent and opaque models achieved average total scores of 53.2 and 55.4, respectively, demonstrating that the SAPIENS model closely replicates human TB anatomy and dissection experience. Significant results were particularly observed in the facial nerve, sigmoid sinus, and cortical mastoidectomy dissections.

This second study further validated the model’s effectiveness in educational programs and surgical skill development. A comparative dissection analysis between the SAPIENS models and a fresh frozen human TB was performed using a specifically designed questionnaire with a 5-point Likert scale. The surgical steps for both the transparent and opaque SAPIENS models were found to be highly similar to those of the cadaveric TB. The transparent and opaque models received total scores of 4/5 and 4.2/5, respectively, in terms of dissection similarity.

Key procedures, such as mastoid region dissection, skeletonization and identification of the tegmen tympani and sigmoid sinus, epitympanic region dissection, posterior tympanotomy, canal wall down simulation, and facial nerve dissection, were all rated as highly comparable to the human TB (Figs. 4 and 5). The only exception was the ossicular chain, which, while anatomically accurate, exhibited greater rigidity and reduced motility compared to human samples. Efforts are currently underway to refine this component of the SAPIENS model to enhance its fidelity to the human ossicular chain.

The reduction in average dissection time of the human temporal bone, after prior dissection of two 3D-printed temporal bone models, suggests an improvement in both anatomical and surgical knowledge of the temporal bone, as well as in surgical skills.

This result is consistent with previous research showing the beneficial effects of simulation-based training using 3D-printed models on surgical skills and self-confidence during procedures [13–19]. The transparent SAPIENS model, which made it easy to observe the arrangement of structures within the temporal bone, was considered the best option for inexperienced surgeons to begin dissection. Its accurate visualization of anatomical structures helps in understanding the location of hidden structures within the temporal bone that are visible in the transparent model (Figs. 2 and 3).

When transitioning to the fresh frozen bone, the memory of dissecting the transparent models allowed for more accurate dissection due to increased confidence in the anatomy, which also reduced dissection times. In contrast, the opaque model closely replicated the texture of a human temporal bone taken from a cadaver, offering a realistic drilling experience with well-pneumatized and compact bone coverage.

Cost-effectiveness, accessibility, enhanced realism, and customization for different surgical scenarios are just a few of the benefits of incorporating 3D-printed temporal bone models into surgical education [14–19].

### Limitations

This study presents some limitations that, nonetheless, offer interesting and realistic directions for future research. While the study group in this preliminary paper is homogeneous in terms of the participants’ experience with temporal bone dissection, its size is relatively small. Ongoing studies aim to include a larger cohort of surgeons with varying levels of experience (inexperienced, moderately experienced, and highly experienced) from different centers or training institutions, in order to optimize and expand upon these initial findings.

The ossicular chain of the SAPIENS 3D-printed model, although identical in structure to the human ossicular chain, was observed to be more rigid and exhibited reduced mobility during manipulation. This is due to the nature of 3D printing. The model was created using a Photon Mono X 4 K desktop printer, which employs a stereolithography (SLA) light-curing system with resin to reproduce all anatomical structures in high detail. The resin used (Anycubic), selected from several available options, was chosen for its closest resemblance to human bone during dissection. However, a primary limitation of SLA printing is that all structures are fabricated from the same material (for more details, refer to Reference 34). As a result, the ossicular chain is made from the same resin used for the entire temporal bone. Additionally, the suspensory ligaments of the ossicular chain and the joints between the incus, malleus, and stapes are also constructed with the same resin, making the ossicular chain stiffer than its human counterpart. Nonetheless, it remains possible to disarticulate the incus from the malleus or dissect the superstructures of the stapes in our 3D-printed model. The use of 3D printers capable of employing different materials for different structures could address this limitation and improve the model’s accuracy in future iterations.

### Summary and outlook

Based on our experience and a review of the literature, we conclude that 3D-printed temporal bone models offer both advantages and disadvantages in the fields of education, surgical otology training, and otologic surgery. The main advantages include:

Improved Visualization: Transparent 3D-printed models can help inexperienced surgeons better understand the complex anatomy of the temporal bone.

Hands-On Learning: Residents and surgeons can use 3D-printed temporal bone models to practice surgical skills without relying on cadaveric specimens.

Preoperative Planning: Surgeons can use 3D-printed models to plan and simulate complex procedures, such as cochlear implantation, improving surgical outcomes and minimizing the risk of complications.

Customization: 3D printing enables the creation of patient-specific models based on medical imaging data, allowing surgeons to replicate malformed or pathological temporal bones. This is especially useful in pediatric cases or instances of middle and inner ear malformations, as it allows surgeons to simulate otologic procedures preoperatively and reduce intraoperative risks in these complex cases.

### Final remarks

We believe that incorporating 3D-printed temporal bone models into surgical training programs can significantly accelerate the learning curve for trainees, improve surgical skills, and enhance surgical outcomes. Additionally, by providing a secure and controlled environment for practice, these models enable trainees to refine their abilities without the pressure of performing otologic procedures on patients without sufficient surgical expertise. Therefore, future research should focus on further validating the effectiveness of these models across different surgical procedures (such as cochlear implantation and labyrinthine dissection) and settings (microscopic vs. endoscopic approaches), as well as exploring additional applications of 3D printing technology in surgical education.

## Conclusion

In conclusion, the integration of 3D-printed temporal bone models into surgical education programs represents a significant advancement in training methodologies. The SAPIENZA 3D-printed temporal bone model can be considered a valuable tool for advancing surgical education due to its anatomical accuracy and its relevance for dissection training.
