# Correction: The SAPIENS 3D-printed temporal bone model: a real tool for advanced otologic surgery education

**DOI:** 10.1007/s00405-025-09531-5

**Published:** 2025-07-23

**Authors:** Giannicola Iannella, Annalisa Pace, Antonio Greco, Armando De Virgilio, Mario Giuseppe Bellizzi, Enrica Croce, Jerome R. Lechien, Antonino Maniaci, Salvatore Cocuzza, Federico Maria Gioacchini, Massimo Re, Andrea Collettini, Lodovica Gatti, Tiziano Perrone, François Simon, Stéphane Gargula, Giuseppe Magliulo

**Affiliations:** 1 Department of ‘Organi di Senso’, University “Sapienza”, Viale dell’Università 33, Rome, 00185 Italy; 2 Faculty of Medicine and Pharmacy, University of Mons (UMons), Mons, Belgium; 3 Department of Otolaryngology, Kore University, Enna, Italy; 4 Department of Medical, Surgical Sciences and Advanced Technologies G.F. Ingrassia, University of Catania, Catania, Italy; 5 Ear, Nose, and Throat Unit, Department of Clinical and Molecular Sciences, Polytechnic University of Marche, Ancona, Italy; 6 Department of Otolaryngology, Civil Hospital of Alghero, Alghero, Italy; 7 Department of Pediatric Otolaryngology, Necker-Sick Children’s Hospital, AP-HP- University of Paris, Paris, France; 8 Department of Otolaryngology, Hôpital Fondation Adolphe de Rothschild, Paris, France

**Correction: European Archives of Oto-Rhino-Laryngology (2025) 282:3051-3063**

10.1007/s00405-024-09199-3

In this article, the author’s name Federico Maria Gioacchini was incorrectly written as Federico Gioacchini.

The original article has been corrected.

